# Risk Factors for *Helicobacter pylori* Infection among a Rural Population in Japan: Relation to Living Environment and Medical History.

**DOI:** 10.2188/jea.13.266

**Published:** 2007-11-30

**Authors:** Yuko Nishise, Akira Fukao, Tatsuya Takahashi

**Affiliations:** 1 Division of Public Health and Preventive Medicine, Department of Diagnostic Information and Socioenvironmental Medicine, Yamagata University School of Medicine.

**Keywords:** *Helicobacter pylori*, risk factors, hepatitis A virus, human, hygiene, endoscopy, gastrointestinal

## Abstract

BACKGROUND: *Helicobacter pylori* infection is related to several gastroduodenal diseases, though the route of transmission remains unclear.

METHODS: A cross-sectional study that included 695 healthy people (males 308, females 387; median age 60 years) participating in a health checkup program in Yamagata Prefecture was conducted. *H. pylori* status was determined in all subjects by evaluation of serum anti-*H. pylori* immunoglobulin G antibody. Antibody against hepatitis A virus was used as a marker of fecal-oral exposure to assess the agreement between *H. pylori* infection and hepatitis A virus infection. Data on other factors known or suspected to be related to infection status were also collected using a questionnaire.

RESULTS: Seroprevalence of *H. pylori* and hepatitis A virus was 60% and 70%, respectively. Kappa values for subjects aged 20-49 and aged 50 or older were 0.07 and 0.02, respectively, and agreement between the presences of both infections was assessed as slight. In the multivariate logistic regression analysis, *H. pylori* infection was significantly associated with availability of a sewage system in childhood (presence [reference], absence [odds ratio (OR)=4.06, 95% confidence interval (CI): 1.36-13.94]) and the number of gastrointestinal endoscopies undergone (none [reference], once [OR=1.64, 95% CI: 0.83-3.27], 2-3 times [OR=3.11, 95% CI: 1.65-5.99], or 4 or more times [OR=3.18, 95% CI: 1.71-6.03]), (p<0.01 for trend).

CONCLUSIONS: Our results suggest that poor hygiene in childhood is related to *H. pylori* infection. The fecal-oral route does not seem to be an important mode of transmission, but the possibility of transmission by gastrointestinal endoscopic examination exists.

*Helicobacter pylori* infection is causally related to chronic gastritis,^1^^,^^2^ peptic ulcer,^3^^,^^4^ and gastric carcinoma.^5^^-^^7^ Although the route of transmission remains unclear, evidence suggests that it is mainly person-to-person^2^^,^^8^^,^^9^ by either oral-oral (through vomitus or possibly saliva) or fecal-oral routes.

The prevalence of *H. pylori* varies both between and within populations, with a generally higher proportion of acquisition in developing countries than in industrialized countries. In Japan, the prevalence of *H. pylori* infection is 10 to 20% in those aged less than 20 years, but up to 70% or greater in those 50 years of age or older.^10^^,^^11^ This prevalence in the older portion of the population is higher than that observed in other developed countries. The age distribution of *H. pylori* infection in Japan, where higher rates of infection occur in earlier birth cohorts, might be due to poor childhood living conditions. The birth-cohort effect would be relevant to childhood acquisition of *H. pylori*.^12^^,^^13^ Although both acquisition and elimination can occur throughout life, the infection rates in adulthood appear to be low in developed countries.^14^ Relatively little is known about factors that might affect acquisition of the infection among adults as compared to children. Although some procedures associated with adulthood, such as gastrointestinal endoscopic examination and dental treatment, are suspected to be risk factors for *H. pylori* infection, few studies have evaluated their impact in population samples.

The aim of the present study is to identify the risk factors and transmission modes for *H. pylori* infections of healthy adults by assessing various factors associated with both childhood and adulthood.

## METHODS

### Study design and population

This cross-sectional study was conducted between October 2000 and July 2001 in a population who participated in a general health checkup program at a single health screening center in Yamagata Prefecture, Japan. During the study period, most participants were inhabitants of N city and T town, which adjoin each other and are located in southern Yamagata. The year 2000 populations of N city and T town were 36,749 and 27,248 inhabitants, respectively. The proportion of participation in the annual checkups was 51.1% in N city and 40.4% in T town. Both areas have similar industrial structures and topographical characteristics. A total of 812 people participated in the health checkup program during the study period. We explained the purpose and procedures of the study to all subjects individually, and 699 (86%) gave their informed consent to participate. After excluding four subjects who had a history of *H. pylori* eradication, the final sample comprised of 695 healthy subjects (308 men and 387 women).

We undertook a serosurvey to determine *H. pylori* and hepatitis A virus status, as well as the risk factors for *H. pylori* infection using a structured questionnaire. Hepatitis A virus status was compared with the seropositivity for *H. pylori*, because hepatitis A virus is well known to be a sensitive marker of fecal-oral exposure.^15^ Serum samples were obtained from all the participants. About half of the questionnaires were completed on the spot, and the rest were returned by mail. Some additional information on sex, age, date of birth and residential region was obtained from medical records taken by nurses at the health- screening center.

### Definition of *H. pylori* infection and hepatitis A virus infection

*H. pylori* status was evaluated by enzyme-linked immunosorbent assay (ELISA) using a high-molecular-weight, cell-associated protein immunoassay kit (Kyowa Medex Co., Ltd., Tokyo, Japan) for anti-*H. pylori* immunoglobulin G (IgG) antibody. The high-molecular-weight, cell-associated protein immunoassay, which in Japan is called Determiner *H. pylori* antibody, provided 91.1% sensitivity and 82.5% specificity, when subjects with intermediate values were excluded.^16^ In our study, intermediate results were included in the positive category because Miwa et al. reported that 83.3% of their patients with intermediate values were positive using the breath test as the gold standard.^16^ Therefore, subjects with an antibody titer higher than the cut-off value of 2.2 were considered to be positive. Anti-hepatitis A virus antibody was assayed by ELISA using an IMxHAVAB kit (Abbott Laboratories, North Chicago, Illinois, USA). Anti-hepatitis A virus antibody levels giving more than 50% inhibition were considered to be positive.

All serum samples were placed in a freezer within 24 h and assayed at a commercial laboratory center.

### Questionnaire

The questionnaire consisted of 36 items related to both the subject’s childhood and present living environments; 10 items related to medical histories, and 22 items to lifestyle. Items related to the living environment during childhood included birth place, the number of siblings, birth order, attendance at kindergarten or nursery school, the number of family members living in the same house, the number of rooms in the house, sharing of living space with others outside the home, type of housing (single-family dwelling, multifamily dwelling, or apartment), use of well water and, if so, its use for drinking, availability of a sewage system, use of a refrigerator, ownership of domestic animals including pets, such as cats or dogs, and farm animals, such as cattle, father’s occupation, and age at graduation from the highest level of school attended. Items related to the present living environment included the number of family members living in the same house, type of housing, the number of rooms in the house, duration of habitation in the present house, marital status (married, divorced, widowed or single), long-term occupation, use of well water and, if so, its use for drinking, availability of a sewage system, and ownership of domestic animals including pets and farm animals. In defining an overcrowded living space, the average number of family members per room both in childhood and at present was calculated by dividing the number of household members by the number of rooms in the house.

Information on medical histories consisted of ABO blood group; frequency of gastric pain (none, infrequent, occasional or frequent); a history of gastric ulcer or duodenal ulcer; previous gastrointestinal endoscopic examinations and, if relevant, their number and the calendar year of the first endoscopic examination; a history of dental treatment and, if relevant, the number of treatments; and the number of health screenings in the past five years.

Selected items related to food intake included rice, *miso* soup, green tea, coffee, pickles, green vegetables, and fruit. The participants were asked to record the average frequencies with which they consumed these food items using five ordered categories. For rice, *miso* soup, green tea and coffee, designations for daily consumption were infrequent, occasional, 1-2 cups per day, 3-4 cups per day, and 5 or more cups per day. For the other food items, categories of intake frequency ranged from infrequent to 1-2 times per month, 1-2 times per week, 3-4 times per week, and every day. Drinking and smoking habits were classified into three categories: never, former and current.

The questionnaires were completed within 30 min on average. To assess the validity of the questionnaire, answers to selected items on the questionnaire were compared with information on 1.1 or more per room (reference 1.0 or less) was negatively associated with *H. pylori* infection with no statistical significance. The number of siblings and birth order were not associated with *H. pylori* infection. As to other factors related to living conditions in childhood, availability of a sewage system in childhood was significantly associated with *H. pylori* infection. Sharing of living space with others outside the home in childhood (OR=0.96, 95% CI: 0.64-1.47), attendance of kindergarten or nursery school (OR=0.88, 95% CI: 0.50-1.55), type of housing in childhood (single dwelling [reference], multifamily dwelling or apartment [OR=0.79, 95% CI: 0.28-2.31]), use of well water for drinking in childhood (OR=1.17, 95% CI: 0.80-1.71), and use of a refrigerator in childhood (OR=1.03, 95% CI: 0.68-1.55) were not significantly associated with *H. pylori* seroprevalence. Among factors related to zoonotic exposure in childhood, pet ownership, especially cat ownership, was significantly associated with *H. pylori* infection. Ownership of pets other than cats (23% of subjects were dog owners, and less than 10% owned other animals) in childhood was not associated with *H. pylori* seroprevalence (data not shown) and ownership of farm animals in childhood (OR=1.14, 95% CI: 0.79-1.63) also was not associated with *H. pylori* seroprevalence. With regard to factors related to the present living environment and lifestyle, no significant relationships were found (data not shown). In relation to the medical history, the history of duodenal ulcer and the number of gastrointestinal endoscopic examinations were significantly associated with *H. pylori* seroprevalence. A positive dose-response relationship was seen between the number of gastrointestinal endoscopies undergone and *H. pylori* infection (p<0.01 for trend). Frequency of gastric pain (none [reference], infrequent or occasional [OR=1.30, 95% CI: 0.91-1.86], frequent [OR=1.18, 95% CI: 0.59-2.38]), history of gastric ulcer (OR=1.19, 95% CI: 0.70-2.09), and calendar year of the first gastrointestinal endoscopic examination were not significantly associated with *H. pylori* infection. Also, ABO blood group (O [reference], A [OR=1.03, 95% CI: 0.68-1.55], B [OR=1.00, 95% CI: 0.62-1.63], AB [OR=0.87, 95% CI: 0.49-1.55]), the number of dental treatments (none [reference], 1-9 [OR=1.32, 95% CI: 0.37-4.59], ten or more [OR=1.64, 95% CI: 0.45-5.78], [p=0.21 for trend]), and the number of health screenings (3 or less [reference], four or more [OR=1.07, 95% CI: 0.88-1.29]) were not significantly associated with *H. pylori* seroprevalence. Factors related to socioeconomic status also were not significantly associated with *H. pylori* seroprevalence.

Multivariate analysis was conducted with the following variables: age, sex, variables with a *p* value <0.1 in age- and sex-adjusted analyses, and variables representing socioeconomic status in childhood and at present, i.e., father’s occupation, subject’s educational level, and subject’s long-term occupation. Due to the high correlation between pet ownership and cat ownership among the total of 12 variables, the latter, which is one potential vector for *H. pylori* transmission,^21^ was included in the final multivariate model. Significant risk factors taking into account other factors were availability of a sewage system in childhood as indicated by presence (reference) and absence in childhood (OR=4.06, 95% CI: 1.36-13.94) and the number of gastrointestinal endoscopies undergone as indicated by none (reference), once (OR=1.64, 95% CI: 0.83-3.27), 2-3 times (OR=3.11, 95% CI: 1.65-5.99), or four or more times (OR=3.18, 95% CI: 1.71-6.03). A positive dose-response relationship was seen between the number of gastrointestinal endoscopies undergone and *H. pylori* infection (p<0.01 for trend), and the same trend (p<0.01 for trend) was seen both in subjects aged 40-59 years, as expressed by none (reference), once (OR=1.74, 95% CI: 0.85-3.66), 2-3 times (OR=2.76, 95% CI: 1.33-5.97), or four or more times (OR=2.87, 95% CI: 1.36-6.37); and those aged 60 years or older expressed as none (reference), once (OR=1.29, 95% CI: 0.51-3.26), 2-3 times (OR=1.66, 95% CI: 0.80-3.45), or four or more times (OR=2.55, 95% CI: 1.24-5.28). In subjects aged 20-49 years, the trend was not estimated due to the insufficient number of exposed subjects.

## DISCUSSION

The age-specific seropositivity percentage in this study was almost the same as that of an earlier study of healthy subjects in Japan,^22^ but lower than that of some studies conducted in the early 1990s.^10^^,^^11^ This difference can be partly explained by a cohort effect or by a difference in study sites. In developed countries, the reported annual *H. pylori* seroconversion rate was found to be less than the seroreversion rate in healthy adults. The seroconversion and seroreversion rates, respectively, were 0.33% and 1.04% per person-year in Australia during 21 years of follow-up,^23^ 0.49% and 1.12% per person-year in the United States during 8.5 years of follow-up,^24^ 1.0% and 1.6% per person-year in Canada in a three-year prospective study,^25^ and 1.0% and 1.5% per year in Japan in an eight-year follow-up study.^26^ (The expression “% per person-year” is directly quoted from the references.)

Hepatitis A virus is known to be spread by fecal-oral contact and has a high incidence in populations with poor hygienic practices and a low socioeconomic level. A positive association between seroprevalence of *H. pylori* and that of hepatitis A virus has been shown,^27^^-^^30^ but this finding has not been consistent.^31^^,^^32^ Luzza et al., in a study of population samples, reported that these two infections did not share any independent risk factors, although there was a statistically significant relationship between seropositivity for both.^33^ In Japan, Furuta et al. reported no statistically significant relationship between *H. pylori* and hepatitis A virus seropositivity rates among subjects who participated in medical checkups.^22^ In contrast, Fujisawa et al. demonstrated a statistically significant association between *H. pylori* and hepatitis A virus seropositivity rates among participants in a health screening program.^34^ However, the latter study did not address some potentially related factors such as childhood conditions or socioeconomic status. In the present study, the concordance between *H. pylori* seropositivity and hepatitis A virus seropositivity was not high, implying that in a high-risk environment for hepatitis A virus infection, the incidence of *H. pylori* infection did not increase accordingly. There is a basic difference in the immune response between these two infections; *H. pylori* seropositivity represents current or recently cleared infection, whereas hepatitis A virus seropositivity represents current or past infection. Although we cannot completely ignore the possibility of fecal-oral transmission of *H. pylori* in a manner that is different from such transmission of hepatitis A virus, we conclude that the fecal-oral route does not play an important role in *H. pylori* transmission.

Some reports have suggested the possibility of water-borne transmission of *H. pylori*. Epidemiologic studies conducted in rural China, Colombia, and Peru showed that water sources are related to the risk of *H. pylori* infection.^35^^-^^37^ H. pylori positivity in drinking water and sewage water samples determined by polymerase chain reaction assays in Peru, Canada, and Japan^38^^-^^41^ provides additional evidence that water-borne transmission might be important, especially in areas with a high prevalence of *H. pylori* infection. *H. pylori* changes to a coccoid form in water,^42^ but it is still unclear whether the coccoid form, which cannot be cultured using ordinary techniques,^43^ can cause infection in humans. In both rural and semi-urban areas in Japan, *H. pylori* has been detected in well water.^40^^,^^41^ If *H. pylori* is common in well water in Japan and has the ability to cause infection, the risk of infection might be higher with increased water intake. The present study found that the use of well water for drinking in childhood did not increase the risk of infection, perhaps indicating that the risk of water-borne transmission via well water is not high in this population. Only exposure to well water used presently for drinking was linked to a weakly increased prevalence (OR=1.56, 95% CI: 0.93-2.69) without statistical significance. We could not determine whether this relationship was an effect of well water-borne transmission in adulthood. Further efforts to detect *H. pylori* in well water samples in this area would clarify the effect of such transmission.

On the other hand, our finding of an increased OR for availability of a sewage system in childhood suggests the possibility of water-borne and/or fecal-oral transmission. Because of the slight agreement between *H. pylori* infection and hepatitis A virus infection, however, we consider that this finding rather reflects an association of *H. pylori* infection with poor hygienic standards. It is difficult to confirm the exact mode of transmission associated with this finding because people living under such conditions carry a higher risk of exposure and have frequent opportunities to acquire the infection due to poor hygienic practices and exposure to contaminated soil, water in the natural environment,^40^ or food that comes into contact with a potential vector such as the housefly.^40^^,^^44^ The law mandating sewage systems was issued in 1900 in Japan, but sewage systems spread gradually. Since the number of regions with modem sewage treatment increased during the childhood of many of the subjects, it is possible that classification of their exposure to adequate sewage systems is imprecise. The subjects who selected the answer affirming the presence of an available sewage system in childhood are those who were relatively young among the subject population (21 to 58 years of age). However, because we could not obtain detailed information on residential regions of the subjects during childhood, it was impossible to confirm whether the answers as to the availability of a sewage system in childhood were correct.

Many epidemiologic studies have assessed a number of potential risk factors for *H. pylori* infection in relation to density of living space or socioeconomic status, especially during childhood. Some indicators of crowded living space have been consistently related to *H. pylori* positivity, including high birth order,^45^ the presence of many siblings,^46^ and domestic overcrowding.^19^^,^^47^ These factors have been considered to represent evidence for person-to-person transmission of *H. pylori* infection. However, adjustment for only age and sex in the present study revealed a negative association between *H. pylori* seropositivity and domestic overcrowding in childhood, and no association was found between *H. pylori* seropositivity and birth order or having many siblings. Such a contradictory result may be due to the difference in housing styles between Japan and the United States,^19^^,^^47^ or due to the fact that our crowding index does not reveal true density because the size of the rooms was not taken into account. In accounting for inconsistent results regarding birth order or the number of siblings, the number of subjects in our study may be insufficient to detect consistent significant relationships. Further studies with a larger sample size are needed.

The present findings demonstrated a strong association between the history of gastrointestinal endoscopic examination and *H. pylori* infection status. There are two possible explanations for this. First, *H. pylori* infection may be transmitted from patient to patient via the endoscope. Second, those who have acquired *H. pylori* infection tend to undergo gastrointestinal endoscopy more often. The latter seems plausible because *H. pylori* infection tends to produce gastroduodenal symptoms or disease. However, we suggest that both possibilities act to increase the prevalence odds. The former possibility can be supported by the following 3 reasons: (1) there are some documented cases of acute *H. pylori* infection occurring via gastrointestinal endoscopes;^48^^-^^51^ (2) a retrospective study has demonstrated acquisition of *H. pylori* via endoscopy;^49^ and (3) a review in Japan has described a syndrome of so-called post-endoscopic acute gastric mucosal lesions.^52^ Naka et al. suggested that the post-endoscopic acute gastric mucosal lesions were caused by infectious agents, especially *H. pylori* cross-infection, transmitted via inadequately disinfected endoscopes.^52^ Investigation of the possible relationship between the post-endoscopic acute gastric mucosal lesions and *H. pylori* began only in the late 1980s, together with studies on effective disinfection methods to eliminate the risk of endoscopic transmission. More than half of our study subjects had undergone their first endoscopic examination before 1990. Consequently, our study subjects might be exposed to insufficiently disinfected instruments. Because of the nature of the cross-sectional design of this study, the positive association between *H. pylori* status and a history of gastrointestinal endoscopy does not necessarily imply a cause-and-effect relationship. However, the significant dose-response relationship between *H. pylori* seropositivity and the number of gastrointestinal endoscopies undergone would suggest a substantial causal relationship between the two. Although the factors leading to persistent *H. pylori* infection are still unclear, even if there are differences either in host susceptibility or strains of *H. pylori*, gastrointestinal endoscopy may serve as a partial mode of *H. pylori* transmission, especially where equipment or techniques for its disinfection are inadequate.

Although information on risk factors was collected retrospectively for this study, the participants had completed their questionnaire before they were informed of their own *H. pylori* status; therefore, recall bias should have been minimal. Also, it is possible factors related to childhood conditions may be underestimated owing to infection in adulthood or elimination of infection, particularly in older people.^14^ This underestimation might contribute to the failure to clarify the most likely means of *H. pylori* transmission, including intra-familial transmission in childhood. Because our study subjects were recruited from participants in a health screening program, any generalization of these results to the normal population should be made with caution.
